# Epidemiology and Associated Risk Factors for Brucellosis in Small Ruminants Kept at Institutional Livestock Farms in Punjab, Pakistan

**DOI:** 10.3389/fvets.2020.00526

**Published:** 2020-09-02

**Authors:** Qudrat Ullah, Tariq Jamil, Falk Melzer, Muhammad Saqib, Muhammad Hammad Hussain, Muhammad Aamir Aslam, Huma Jamil, Muhammad Amjad Iqbal, Usman Tahir, Shakeeb Ullah, Zafar Iqbal Qureshi, Stefan Schwarz, Heinrich Neubauer

**Affiliations:** ^1^Institute of Bacterial Infections and Zoonoses, Friedrich-Loeffler-Institut, Jena, Germany; ^2^Faculty of Veterinary and Animal Sciences, Gomal University, Dera Ismail Khan, Pakistan; ^3^Department of Theriogenology, Faculty of Veterinary Science, University of Agriculture, Faisalabad, Pakistan; ^4^Institute of Microbiology and Epizootics, Freie Universität, Berlin, Germany; ^5^Department of Clinical Medicine and Surgery, Faculty of Veterinary Science, University of Agriculture, Faisalabad, Pakistan; ^6^Independent Researcher, Bardia, NSW, Australia; ^7^Institute of Microbiology, Faculty of Veterinary Science, University of Agriculture, Faisalabad, Pakistan; ^8^Veterinary Research Institute, Lahore, Pakistan; ^9^Livestock and Dairy Development, Government of Punjab, Lahore, Pakistan

**Keywords:** sheep, goats, brucellosis, risk factors, Pakistan

## Abstract

Brucellosis is reportedly endemic in ruminants in Pakistan. Both *Brucella abortus* and *B. melitensis* infections have been decumented in domestic animals and humans in the country. This study aimed to identify the burden of anti-*Brucella* antibodies in small ruminants as well as associated potential risk factors with its occurrence at nine institutional livestock farms in Punjab, Pakistan. The sera collected from equal number of sheep and goats (500 from each species) were screened by indirect-ELISA for anti-smooth-*Brucella* antibodies followed by a serial detection by real-time PCR. Overall, 5.1% (51/1000) seropositivity was registered corresponding to 5% (25/500) prevalence in goats and 5.2% (26/500) in sheep. *Brucella*-DNA could not be detected in any of the tested sera by real-time PCR. Multiple logistic regression model indicated that farm location (OR 34.05), >4 years of age (OR 2.88), with history of reproductive disorders (OR 2.69), and with BCS of ≤ 3 (OR 12.37) were more likely to test positive for brucellosis at these farms. A routine screening, stringent biosecurity, and quarantine measures are warranted for monitoring and eradication of the infection. Similarly, isolation and molecular investigation of the etiologic agent(s) are needed to understand the relationship of epidemiology and out-breaks of brucellosis in the country.

## Introduction

Brucellosis is a bacterial zoonosis with worldwide distribution, which is caused by bacteria of the genus *Brucella*. This genus comprises; *B. melitensis, B. abortus, B. suis, B. canis, B. ovis*, and *B. neotome* (classical *Brucella* species), *B. ceti* and *B. pinipedialis* from marine mammals, *B. microti* from voles, *B. inopinata* from human females, *B. papionis* from baboons and recently *B. vulpis* from red foxes (1–6). Based upon host preference; *B. abortus* predominantly infects bovines, *B. melitensis*

small ruminants, *B. canis* dogs, *B. suis* pigs, and *B. ovis* rams, however, infection in non-prefered hosts is transmissible (7–9). In developing countries, a higher prevalence rate is observed where it causes abortion and retention of fetal membranes (10). The infection may stay undiagnosed due to its asymptomatic form and the infected animals may conceive subsequently, but remain carriers for their life. The infection is of economic importance, especially in developing countries (11). Direct or indirect contact with infected animals and consumption of contaminated raw milk and products are the main routes of transmission, respectively, in animals and humans (12). Brucellosis is an established occupational health hazard (13–16). Diagnosis remains a challenge and is based primarily on serology [e.g., Rose Bengal Test (RBT) and Milk Ring Test (MRT)]. Molecular detection of *Brucella*-DNA (e.g., PCR) in clinical/biological samples, is coupled with serology to identify the etiology precisely where necessary. The bacterial isolation is a gold standard for the diagnosis, but requires specific growth conditions. Moreover, owing to fastidious nature of the organism (*B. abortus* for one), the turn-around-time for the samples is beyond a week. Vaccination is recommended but practiced mostly in elite herds in developing countries including in Pakistan (17). Treatment of brucellosis in ruminants is also not very popular in the country hence, test and slaughter/culling policy remains a sole solution for eradication of the infection in farm animals.

Pakistan is an agriculture-based country in south-Asia, where livestock plays a vital role in the national economy. The total livestock population in the country is 142.8 millions, where small ruminants (sheep and goat) share 80.27 million heads (18). In the past, brucellosis has been reported in both large and small ruminants in Punjab, Pakistan (19–23). This study was aimed to ascertain the current status of brucellosis in small ruminants at institutional livestock farms located in Punjab. Additionally, we determined the risk factors associated with the occurrence of the disease.

## Materials and Methods

**Figure 1:**
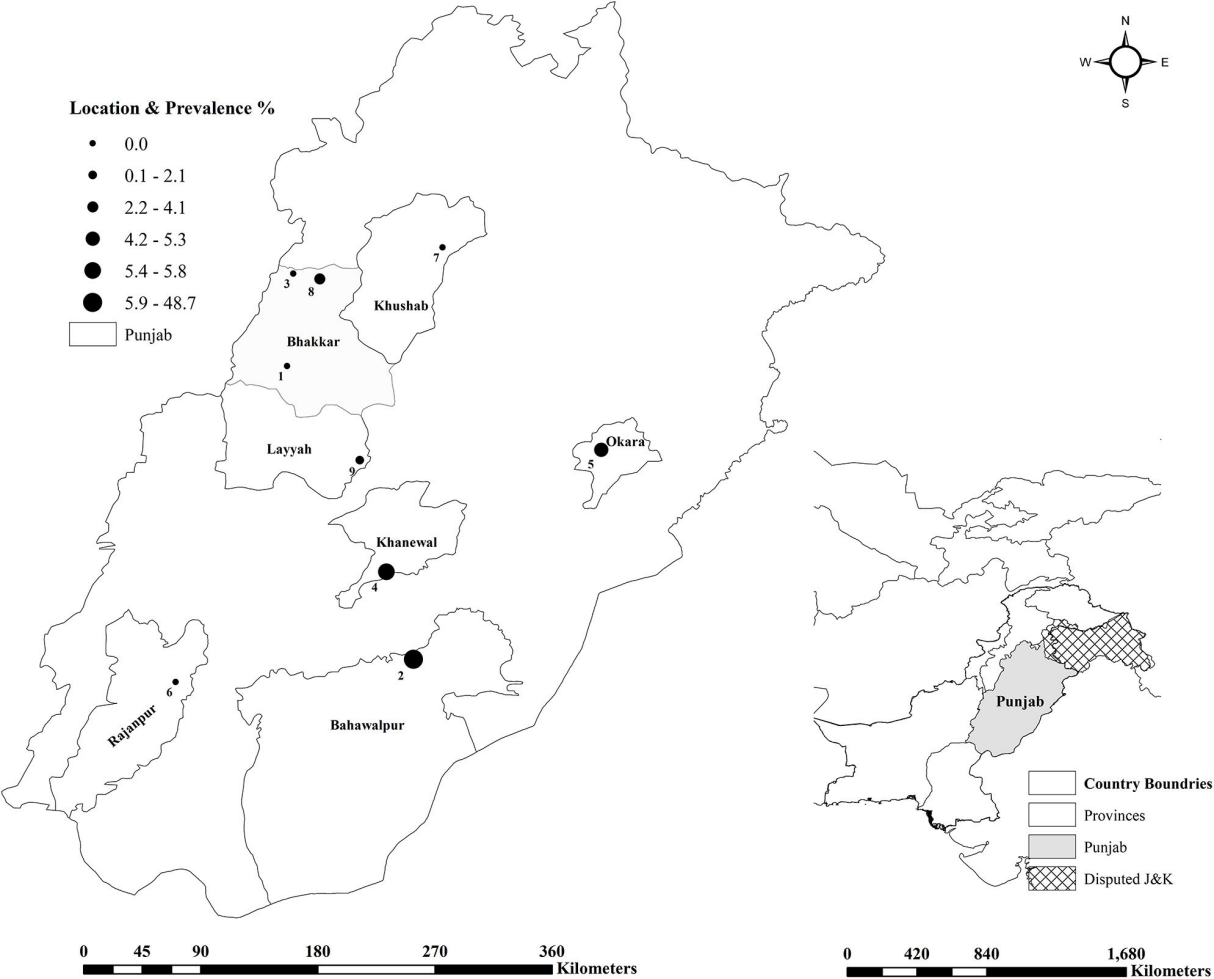
Frontiers Media SA remains neutral with regard to jurisdictional claims in published maps and institutional affiliations. Gegraphical representation of the small ruminant farms tested for brucellosis in Punjab, Pakistan.

A total of 1,000 sera (500 each from sheep and goats) were collected from nine different institutional livestock farms maintained under the Livestock and Dairy Development Department (L&DD), Government of Punjab, Lahore, Pakistan (Figure 1) (24). The sample size was calculated for an estimated disease prevalence of 50% at a 95% confidence interval, and 5% desired absolute precision (Table 1) (25). A minimum of 384 samples from each species were required by this method. The sample size was further inflated to accommodate for the potential losses during the transportation. The final sample size was proportionally allocated to each farm according to the population of the animals at each farm. Available identification record was used at each farm, to randomly select animals by using a random number generator and to collect the animal level data. Individual animals were restrained and blood was collected in a 9 mL vacutainer tube without anticoagulant through the jugular vein. No animals were harmed during this process. The animals had no prior history of brucellosis vaccination.

**Table 1:** Seroprevalence in small ruminants of Punjab, Pakistan.

**Farm^**a**^**	**Goats**^****b****^		**Sheep**^****c****^		**Total**	
	**Pos./Tested**	**Prev.%(95% CI)**	**Pos./Tested**	**Prev.%(95% CI)**	**Pos./Tested**	**Prev.%(95% CI)**
1	0/0	-	0/41	0 (0–8.6)	0/41	0 (0–8.6)
2	0/0	-	18/37	48.7 (31.9–65.6)	18/37	48.7 (31.9–65.6)
3	0/0	-	0/22	0 (0–15.4)	0/22	0 (0–15.4)
4	13/203	6.4 (3.5–10.7)	1/40	2.5 (0.1–13.2)	14/243	5.8 (3.2–9.5)
5	7/44	15.9 (6.6–30.1)	0/88	0 (0–4.1)	7/132	5.3 (2.2–10.6)
6	0/43	0 (0–8.2)	0/9	0 (0–33.6)	0/52	0 (0–6.8)
7	0/0	-	0/45	0 (0–7.9)	0/45	0 (0–7.9)
8	0/0	-	6/145	4.1 (1.5–8.8)	6/145	4.1 (1.5–8.8)
9	6/210	2.9 (1.1–6.1)	0/73	0 (0–4.9)	6/283	2.1 (0.8–4.6)
**Total**	**26/500**	**5.2 (3.4–7.5)**	**25/500**	**5 (3.3–7.3)**	**51/1,000**	**5.1 (3.8–6.7)**

a The seroprevalence varied significantly among sampled farms; χ^2^ = 159.281, p < 0.001.

b The seroprevalence in sheep varied significantly among sampled farms; χ^2^ = 163.790, p < 0.001.

c The seroprevalence in goats varied significantly among sampled farms; χ^2^ = 15.530, p = 0.001.

Sera were screened by ID Screen® Brucellosis Serum Indirect Multi-species (IDVet, Grabels, France), an indirect-ELISA for detection of anti-smooth-lipopolysaccharide (LPS) (*B. abortus, B. melitensis*, and *B. suis*). The samples were tested at the National Reference Laboratory (NRL) for brucellosis, Friedrich-Loeffler-Institut (FLI), Jena, Germany as per manufacturer's recommendations. DNA was extracted from sera by using the High Pure Template Kit (Roche, Rotkreuz, Switzerland) and molecular detection was serially done by real-time PCR as described by Probert et al. (26). The DNA extraction was run along with *E. coli* controls. The real-time PCR was run along with *B. abortus* (ATCC 23448) and *B. melitensis* (ATCC 23456) as positive controls. In tandem with positive controls, nuclease-free water was run as negative control (NTC).

Brucellosis prevalence at species level was calculated by dividing the number of positive animals (numerator) by the total number of animals sampled (denominator). The statistical analysis was performed in two parts. In the first part, univariate and multivariate analysis were conducted to determine the association of the risk factors with the seroprevalence. The univariate analysis was conducted for farm related and animal level variables. Seroprevalence of brucellosis was considered as an outcome or dependent variable while biological plausible variables [e.g., farm location, species, sex, age/parity status, breed, history of reproductive disorders, and body condition score (BCS)] were considered as explanatory or independent variables. A *p* ≤ 0.05 was considered as a level of significance. A backward stepwise approach was used for the binary logistic regression analysis (27). Nagelkerke *R*^2^ (NR^2^) and Hosmer and Lemeshow test (HLT) were used to assess the model-fitness. The statistical analysis was conducted using the IBM SPSS Statistics (IBM Corporation, Armonk, New York, USA).

The second part of statistical analysis was performed using R software and each of the variable was tested one by one alone in a mixed effect model approach with “farm” variable as random factor and using “lmer” function from lme4 package, and logistic binary model function (28). The results of these models showed that five variables were significantly associated with seroprevalence of brucellosis, i.e., species, age, parity status, reproductive disorders, and body condition score (see Table 4). To check if any of these variables showing significance association were confounded, all the five variables were tested in one single model and stepwise backward regression was performed (i.e., least significant variables were taken out in the next model). After running the model, collinearity and confounding behavior was tested by determining variance inflation factor using “vif” function from “car” package. Those variables were taken out of the model which showed high *p*-value and high variance inflation factor. In the next model if the *p*-value and variance inflation factor of the other remaining variables changed by a factor of 20%, then the taken-out variable was considered to be confounded with other variables. The maps were generated by using ArcGIS version 10.5.1 (ESRI, Redlands, CA, USA).

## Results

Anti-*Brucella* antibodies were detected in 51 (5.1%, CI 3.8–6.7) samples from sheep and goats. The farm-herd based and univariate analysis showed the seroprevalence almost identical in goats (5.2%) and sheep (5.0%), *p* = 0.886 (Tables 1, 2). Seropositive animals were detected at the five of nine sampled farms, and the prevalence varied from 2.1% (Farm 9) to 48.7% (Farm 2), *p* < 0.001. In goats, the highest seroprevalence was recorded in the small ruminants at Farm 5 (15.9%) and the lowest at the Farm 9 (2.9%), *p* = 0.001. In sheep, the seropositivity ranged from 2.5% (Farm 4) to 48.7% (Farm 2), *p* < 0.001 (Figure 1). None of the samples contained *Brucella* DNA as confirmed by negative real-time PCR results.

**Table 2:** Univariable analysis of the seroprevalence of brucellosis in small ruminants sampled from nine institutional livestock farms of Punjab, Pakistan.

**Variable**	**Category**	**Pos. /tested**	**Prev. % (95% CI)**	**Odds ratio**	**95% CI**	*p*-value^*^
Farm	Farm 2	18/37	48.7 (31.9–65.6)	25.7	12.84–55.52	<0.001
	Others	33/963	3.4 (2.4–4.8)	Ref	-	
Species	Sheep	26/500	5.2 (3.4–7.5)	1.042	0.593–1.831	0.886
	Goats	25/500	5 (3.3–7.3)	Ref	-	
Sex	Females	47/893	5.3 (3.9–6.9)	1.43	0.51–4.05	0.5
	Males	4/107	3.7 (1–9.3)	Ref	-	
Age	Above 4Y	35/440	7.9 (5.6–10.9)	2.94	1.60–5.38	<0.001
	Below 4Y	16/560	2.9 (1.6–4.6)	Ref	-	
Parity Status	Multiparous	40/594	6.7 (4.9–9.1)	2.59	1.31–5.12	0.006
	Nulli/Primi	11/406	2.7 (1.4–4.8)	Ref	-	
Breeds	Buchi	18/37	48.7 (31.9–65.6)	26.7	12.84–55.52	<0.001
	Others	33/963	3.4 (2.4–4.8)	Ref	-	
Reproductive disorders	Yes	25/178	14.0 (9.3–20.0)	5.00	2.81–8.89	<0.001
	No	26/822	3.2 (2.1–4.6)	Ref	-	
BCS	< underline < >3	34/172	19.8 (14.1–26.5)	11.74	6.39–21.62	<0.001
	>3	17/828	2.1 (1.2–3.3)	Ref	-	

* *Statistical value of significance: p ≤ 0.05*.

The univariable analysis indicated that sheep at Farm 2 were significantly (*p* < 0.001) more likely to test positive for anti-*Brucella* antibodies (OR 25.7, CI 12.84–55.52). In females, the seropositivity (5.3%) and odds for testing positive (OR 1.43, 0.51–4.05) were higher as compared to males (3.7%), *p* = 0.5. The small ruminants; above 4 years of age (7.9%, OR 2.94 CI 1.60–5.38), of multiparous status (6.7%, OR 2.59 CI 1.31–5.12), belonging to Buchi breed (48.7%, OR 26.7 CI 12.84–55.52), with history of reproductive disorders (13.6%, OR 3.19 CI 1.29–7.95) and having BCS ≤ 3 (19.8%, OR 11.74 CI 6.39–21.62) were found significantly (*p* < 0.05) more likely to test seropositive (Table 2).

The multivariable analysis indicated that small ruminants; kept at Farm 2 (OR 34.05 CI 13.47–86.10), above 4 years of age (OR 2.88 CI 1.39–5.94), with history of reproductive disorders (OR 2.69 CI 1.33–5.42), and BCS ≤ 3 (OR 12.37 CI 5.98–25.57) were significantly (*p* < 0.01) more likely to test positive for anti-*Brucella* antibodies (Table 3). The values of Nagelkerke *R*^2^ (0.407) and Hosmer and Lemeshow test (Ci-square value; χ2 = 3.092, *p* = 0.543) indicated that it was a reasonable model to predict seroprevalence of brucellosis at the sampled farms.

**Table 3:** Multivariable analysis of the seroprevalence of brucellosis in small ruminants sampled from nine institutional livestock farms of Punjab, Pakistan.

**Variable**	**Exposure variable**	**Comparison**	**OR**	**95%CI**	*p*-value^*^
Farm	Farm 2	Others	34.05	13.47–86.10	<0.001
Age group	>4 years	<4 years	2.88	1.39–5.94	0.004
Reproductive disorders	Yes	No	2.69	1.33–5.42	0.006
BCS	< underline < >3	> 3	12.37	5.98–25.57	<0.001

*Model Fit: Nagelkerke R^2^ = 0.407, Hosmer and Lemeshow Test (χ^2^ = 3.092, p = 0.543)*.

* *Statistical value of significance: p ≤ 0.05*.

In the second part of statistical analysis, using mixed effects model approach while testing each variable one by one in each model, the following were significant, i.e., species, age, parity status, reproductive disorders, and body condition score while sex and breed were non-significant (Table 4). Using backward regression analysis, testing all these five significant variables together, species and body condition score were found significant while age, parity status, and reproductive disorders were non-significant, with age showing least significant *p*-value (0.82) and high vif value (3.50) (Table 5). Variable “age” was taken out in the next model, and species, parity status, and body condition score were significant while reproductive disorders was non-significant (0.33) in this model and all variables showed lower vif values. Variable “reproductive disorders” was taken out in the next model, and all the remaining three variables (i.e., species, parity status, and body condition score) were significant and displayed low vif values. Low vif values in the last model pointed out that all the three variables were not confounded (Table 5).

**Table 4:** Each independent variable was tested separately in Mixed effect logistic regression model with farm as random factor.

**Dependent variable**	**Model Sr. No**	**Independent variable**	**Estimate**	***z*-value**	*p*-value^*^
*Brucella-*iELISA outcome	1	Species	−2.546	−2.903	0.003
	2	Age	0.4379	2.563	0.01
	3	Sex	−0.1153	−0.203	0.83
	4	Parity	−1.1371	−3.033	0.002
	5	Breed	−0.1660	−0.995	0.31
	6	Reproductive disorder	0.3344	2.814	0.004
	7	BCS	−2.8795	−7.739	1e−14

* Statistical value of significance: p ≤ 0.05.

**Table 5:** Stepwise backward regression models with starting model containing five independent variables and farm as random factor^*^.

**Dependent variable**	**Model Sr. No**	**Independent variables tested together in one model**	*p*-values^*^	**Variance inflation factor (vif) value**
*Brucella-*iELISA outcome	1	Species	0.003	1.01
		Age	0.82	3.50
		Parity status	0.16	3.44
		Reproductive disorders	0.35	1.07
		BCS	6.63e−14	1.06
	2	Species	0.003	1.01
		Parity status	0.005	1.09
		Reproductive disorders	0.33	1.06
		BCS	5.48e−14	1.04
	3	Species	0.002	1.01
		Parity status	0.001	1.03
		BCS	1.83e−14	1.04

* Variable showing least significance and high variance inflation factor (vif) value were taken out in next model; (Statistical value of significance: p ≤ 0.05).

## Discussion

Brucellosis remains an endemic infection in livestock in Pakistan (17, 29). Serology is a preferred and handy choice for diagnosis of brucellosis. ELISA is a sensitive test and is useful for diagnostic screening on larger scale but is unable to differentiate precisely between vaccinated and infected animals (30, 31). Molecular biological tests e.g., PCR, focus on the presence of DNA in the sample and are potentially able to differentiate the vaccine and field strains of *Brucella* (32). Real-time PCR can even detect and differentiate at lower amounts of DNA in a clinical sample when compared to conventional PCR. However, it requires the presence of bacterial DNA in the sample, which may not be present at every time during and after an infection and might be affected by laboratory procedures (33). Hence, a proper validation process is needed for every test. We used indirect-ELISA as a single screening test and real-time PCR for confirmation of the etiology.

Among variables, the odds for testing positive varied significantly depending upon the farm location and were significantly higher in the animals kept at Farm 2 (Tables 1, 3). These findings are supported by previous reports (20, 22, 34). This could be related to the environmental factors including herd management system at these farms. Furthermore, small ruminants had a close contact with bovines at Farms (2, 5, 6, 7, and 8), where brucellosis was reported previously (21, 23, 35). Moreover, common grazing and watering areas, use of brucellosis positive males for breeding and introduction of new animals without testing could be the factors responsible for brucellosis incidence at these locations (36, 37).

Age (>4 years) and parity status (multiparous) were found significantly associated (*p* < 0.05) with higher odds as compared to younger (<4 years) and null/primiparous (≤ 1 parturited) animals, respectively. Furthermore, age was also found significantly associated (*p* < 0.05) with seroprevalence (OR 2.88) by multivariate analysis (Table 3). A similar trend was reported in both sheep and goats with significant association (21, 38), non-significant association (22), and without determination of association (39, 40). This may be ascribed to increased frequency of contact with other animals with respect to age, higher coital chances, and sexual maturity as compared to younger animals (12, 41).

Reproductive disorders showed significant association (OR 2.69, *p* = 0.006) with brucellosis in the current study (Tables 2, 3). It is understandable as late abortion and retention of fetal membranes are characteristic signs of brucellosis. These findings are supported by similar results reported previously by others investigators (19, 34, 42). However, a non-significant association (*p* > 0.05) in sheep has also been documented (22). Furthermore, animals having BCS ≤ 3, were more likely to test positive (OR 12.37, *p* < 0.001) in our study which is concordance with findings of Ethiopian workers (43). A possible reason could be the higher susceptibility of animals already infected with brucellosis to other infections or the loss in BCS caused by the brucellosis itself.

## Conclusion and Recommendations

In conclusion, we found anti-*Brucella* antibodies in sheep and goats at these livestock farms in Punjab, Pakistan. Farm location, age, and species of the animals, history of reproductive disorders and BCS were found to play a significant role for brucellosis seropositivity in these animals. Although vaccination is recommended and treatment is possible for brucellosis, they are not considered safe for human health, hence regular screening and culling of the reactor animals remain the only choice to monitor and eradicate brucellosis. Introduction of the new stock at these farms should be carried out only after screening and quarantine. Furthermore, farm workers should be advised to adopt protection measures as a routine. Abortion at these farms should not go unnoticed and must be investigated to confirm its cause to adopt recommended control measures. If abortions occur, disinfection of the area should be ensured along with strict biosecurity measures to restrict chances of dissemination of infection through the dogs, cats, other domestic animals, visitors, and farm equipment/supply movement. Standardization and validation of the diagnostic tests are required based on the local conditions. Isolation and molecular investigations of the etiological agents might be helpful for future understanding of the epidemiology of the infection and the relationship of the outbreaks.

## Data Availability Statement

All datasets generated for this study are included in the article/supplementary material.

## Ethics Statement

Blood and serum samples were collected as per bio-safety, ethical, and animal welfare guidelines defined by Research Board of the University of Agriculture, Faisalabad, Pakistan (letter No. 3253/ORIC, dated: 25.11.2015). Permission was granted by the Livestock and Dairy Development Department (LNDD), Government of Punjab, Pakistan to collect samples at the farms (vide letter No. SO (I&C)/L&DD/2-6/2016, dated: 15.02.2016).

## Author Contributions

QU and TJ: Conceptualization. FM and MS: methodology. MH and MA: formal analysis. TJ and QU: investigation. UT, MI, and QU: data curation. TJ: writing-original draft preparation. SU, ZQ, HJ, SS, and HN: writing-review and editing. All authors contributed to the article and approved the submitted version.

## Conflict of Interest

The authors declare that the research was conducted in the absence of any commercial or financial relationships that could be construed as a potential conflict of interest.
